# “Boot Sign” of Anterior Femoral Condylar Resectional Shape during Total Knee Arthroplasty Is More Frequent in Asian Patients

**DOI:** 10.3390/jpm13121684

**Published:** 2023-12-04

**Authors:** Seong Hwan Kim, Yong-Beom Park, Suk Ho Baek, Jeuk Lee, Han-Jun Lee

**Affiliations:** 1 Department of Orthopedic Surgery, Chung-Ang University Hospital, Chung-Ang University College of Medicine, 102 Heukseok-ro, Dongjak-gu, Seoul 06973, Republic of Korea; 2 Department of Orthopedic Surgery, Chung-Ang University Gwangmyeong Hospital, Chung-Ang University College of Medicine, 110 Deokan-ro, Gwangmyeong-si 14353, Republic of Korea; 3 Madisesang Hospital, 890 Dongil-ro, Jungnang-gu, Seoul 02038, Republic of Korea

**Keywords:** femoral rotation angle, symmetric morphology of femoral condyle, total knee arthroplasty, anterior resection surface, grand-piano sign, boot sign

## Abstract

Purpose: There is lack of intraoperative consensus on the distal femur anterior resected surface shape that allows reliable rotational alignment assessment during total knee arthroplasty (TKA). We aimed to evaluate the ratio and prevalence of anterior femoral resection surface intraoperatively. Materials and Methods: The study included 234 osteoarthritis patients with varus knees and not valgus knees or deformities. After conventional medial parapatellar approach, measured resection technique based on the mechanical axis of the femur and preoperative TEA-PCA angle on CT with anterior reference was used among all the patients. The anteroposterior (AP) lengths after distal femoral resection were measured as the femoral lateral AP (FLAP) and femoral medial AP (FMAP) lengths. Based on the medial (MD) and lateral condyle (LD) vertical distance ratios of the femur anterior resected surface, the groups were classified into “boot sign”, “grand-piano”, and “butterfly sign” groups. For comparison of the mean values, the data were assessed for normality with the Shapiro–Wilk test. One-way ANOVA with post hoc analysis using Tukey’s honestly significant difference (HSD) test was used to compare the mean values among the groups. The correlations between the MD/LD and variables were analyzed using the Pearson correlation coefficient. Linear regression analyses were used to find the associated factors to the anterior femoral resection surface shape. Results: Mean intraoperative femoral rotation and distal femoral cutting angles were 4.9° ± 1.2 and valgus 5.0° ± 0.7, respectively. Mean FLAP was 52.9 ± 4.2 mm. Mean MD/LD (0.61 ± 0.13) was lower than that of typical “grand-piano sign”. The morphological shape incidence of the “boot sign” was 62.4%. In the “boot sign” group, the FLAP was found to be smaller than that in the other groups (52.4 ± 4.2 vs. 53.7 ± 4.2 vs. 54.9 ± 2.7; *p* = 0.02), while the intraoperative femoral rotation angle was found to be larger than in the other groups (5.0 ± 1.2 vs. 4.6 ± 1.1 vs. 4.7 ± 1.2; *p* = 0.039). The MD/LD-associated factors were FLAP, intraoperative femoral rotation, and distal femoral cutting angles (R^2^ = 0.268). Conclusion: The femur anterior resection surface shape in TKA was found in the “boot sign” rather than the “grand-piano sign” in Korean ethnics owing to an asymmetric morphology of femoral condyles. Ethnic differences, including distal femoral morphology, should be considered for assessment of the femoral rotation angle using the femur anterior resection surface shape.

## 1. Introduction

The rotational alignment of the femoral component in total knee arthroplasty (TKA) has been reported to be an important factor in patellofemoral and tibiofemoral knee kinematics [1,2,3,4,5]. Excessive internal rotation of the femoral component with respect to the femoral posterior condylar axis (PCA) can lead to knee pain and stiffness, which negatively affects clinical outcomes [2,6,7,8,9]. Several axes, including the transepicondylar axis (TEA), PCA, and the anteroposterior (AP) axis (Whiteside line), are commonly used in an attempt to achieve the correct femoral component rotation [10,11,12,13]. However, controversies remain about which axis should be used for correct rotational alignment due to the fact that each axis has pros and cons, which leads to various contraindications for the use of each axis.

To overcome the lack of consensus concerning the correct component rotation, several attempts have been made to determine preoperative femoral component rotation, each with limitations. When using CT, errors could be made, as it fails to detect the cartilaginous status of the posterior femoral condyles [14,15]. To overcome this problem, MRI can be used; however, this has not yet been fully validated, and is difficult to perform preoperatively for TKA due to cost [15,16,17,18]. If navigation-guided TKAs were performed, the anatomical landmark identification instrument would be placed on the cartilage of the posterior femoral condyles, which could affect the femoral rotation angle for intact and non-impaired cartilage.

The shape of the anterior resected surface of the femur, the so-called “grand-piano sign,” has been used as a landmark to assess the correct rotational alignment during TKA [11,12,13,19,20,21]. Previous simulated anatomical studies have reported morphologic patterns of the anterior femoral condylar resection surface as a reliable intraoperative reference to assess femoral rotational alignment in TKA [19,20,21]. In a previous study, these morphologies were defined using the ratio of the medial and lateral anterior femoral resection surfaces, typically 0.65 to 0.69 as the “grand-piano sign”, 0.5 to 0.55 as the “boot sign”, and 0.8 to 0.98 as the “butterfly sign” [21]. However, only a few simulation studies have reported the shape of the femoral anterior cutting plane using computed tomography (CT) or cadavers. Further, there is a lack of intraoperative consensus on the “real” ratio of the anterior resected surface. In addition, several studies have reported different distal femoral morphology among ethnicities [22,23]. Asians have a narrower distal femur compared to Caucasians.

This study aimed to investigate the morphological shape of the anterior femoral condylar resection surface intraoperatively after bone resection during primary TKA. It is hypothesized that the shape would be close to the “boot sign” rather than the “grand-piano sign” in East Asian ethnics.

## 2. Materials and Methods

This retrospective cohort study enrolled 337 patients who underwent primary TKA between 2018 and 2020. Patients with osteoarthritis (OA) with varus knees were included in this study. Patients with valgus knees, previous surgery history, bony defects, a flexion contracture of more than 30° requiring an additional distal femoral cut of more than 9 mm, or those for whom femoral epicondyles could not be seen accurately on CT were excluded from the study. Finally, 234 patients were included in the study. Health conditions and physiological condition (underlying disease) of every patient enrolled in this study were evaluated to estimate operability before surgery, and no problems were found.

### 2.1. Radiographic Measurements

**Figure 1:**
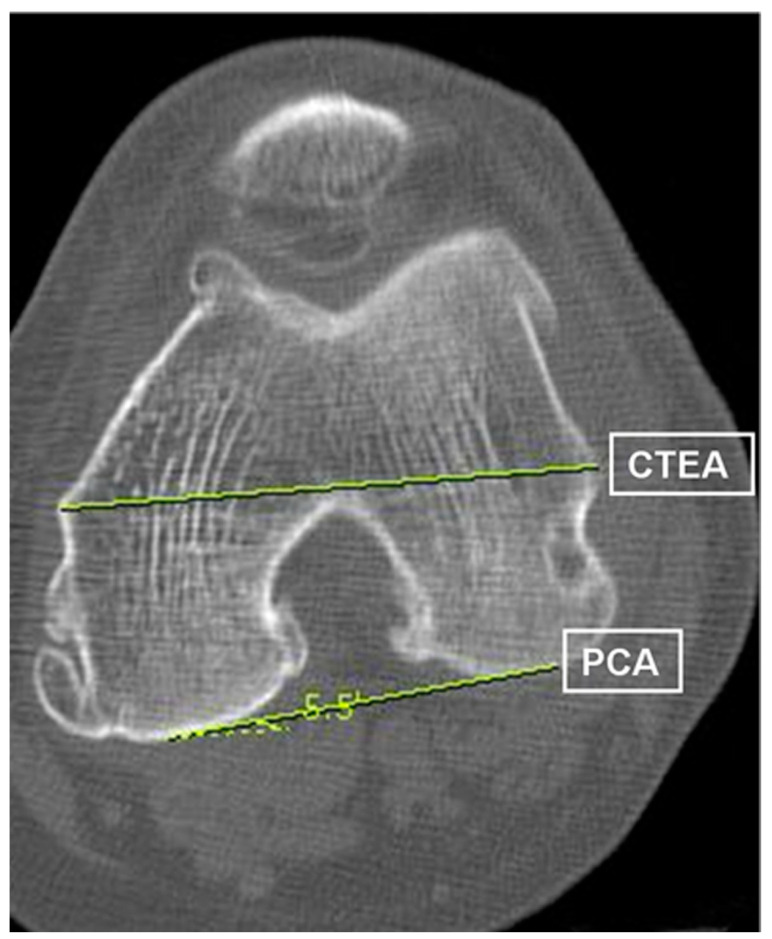
Angles between clinical transepicondylar and posterior condylar axes measured on the CT images are defined as preoperative femoral rotation angle on CT (TEA-PCA). CT, computed tomography.

The mechanical hip–knee–ankle axis angle (HKA), mechanical medial proximal tibial angle (MPTA), anatomical lateral distal femoral angle (LDFA) femoral coronal angle, and sagittal bowing angle were measured on a simple radiograph [3,24,25,26]. The varus alignment was set to positive for HKA. Preoperative radiographic measurements of the femoral rotation were performed using CT with slice thickness of 1.2 mm. Transverse images through the most prominent points of the medial and lateral femoral epicondyles were used for the measurements. The TEA was defined as a line connecting the most prominent points of the medial and the lateral epicondyles. The PCA was defined as the line connecting the most prominent points of the medial and lateral femoral posterior condyles [16,24]. The angles between the two axes measured on the CT images were defined as the preoperative femoral rotation angle on CT (TEA-PCA) (Figure 1). All measurements were performed using a picture archiving and communications system (PACS; General Electric, Chicago, IL, USA).

### 2.2. Surgical Technique

**Figure 2:**
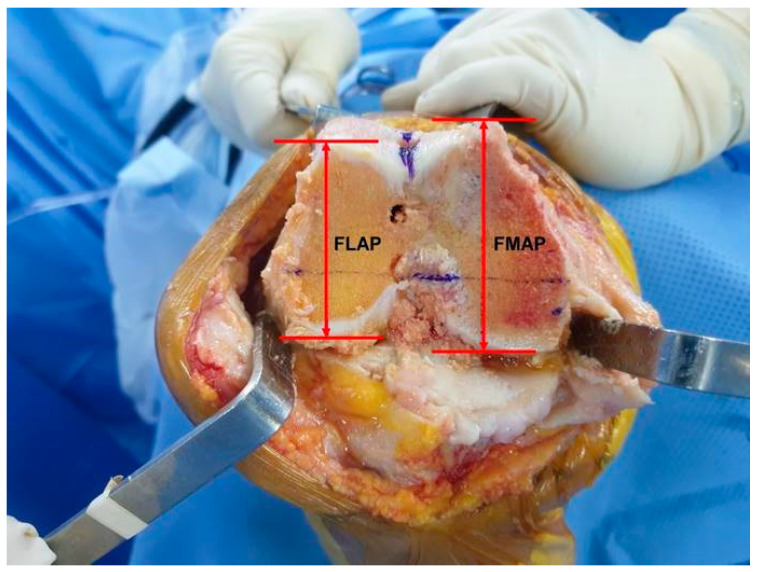
Intraoperative measurements of the anteroposterior length of the lateral femoral condyle (FLAP) and anteroposterior length of the medial femoral condyle (FMAP).

A conventional medial parapatellar approach was performed by sacrificing the posterior cruciate ligaments [24,27]. Distal femoral resection was performed with a distal resection thickness of 9 mm based on the mechanical axis of the femur and the distal femoral cutting angles were recorded. After distal femoral resection, the femoral lateral AP (FLAP) and femoral medial AP (FMAP) lengths were outlined on the surface of the distal femoral resection and measured intraoperatively using a Vernier caliper (Figure 2). The difference between FLAP and FMAP (dFAP), defined as FMAP minus FLAP, was recorded. The intraoperative femoral rotation angle was determined by preoperative TEA-PCA angle on CT combined with the consideration of the lateral femoral remnant cartilage thickness and mediolateral gap balancing [24]. The medial and lateral flexion gap differences measured as less than 2 mm using a laminar spreader were accepted for the gap measurement [24,27]. The anterior referenced TKAs were performed for this study. All prostheses were fixed with cement.

### 2.3. Measurement of the Intraoperative Anterior Resected Surface Morphology of the Distal Femur

**Figure 3:**
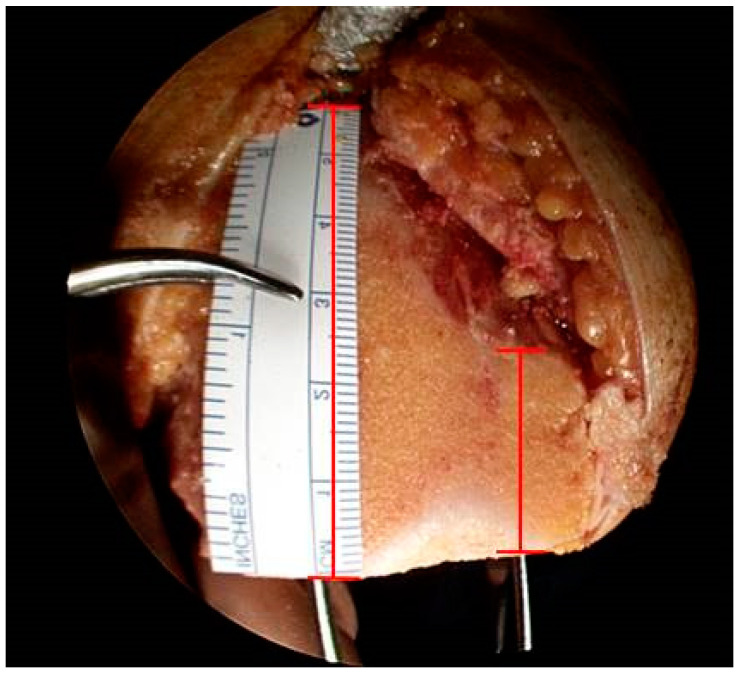
Medial distance (MD) and lateral distance (LD) are measured and the ratio (MD/LD) is calculated after bone resection.

**Figure 4:**
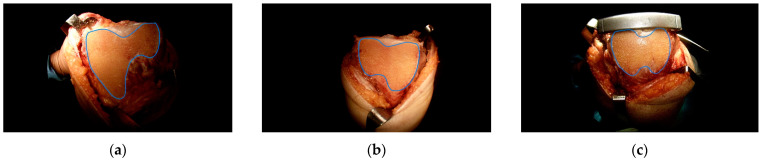
Shapes of the anterior femoral surface: (**a**) “boot sign”, (**b**) “grand-piano sign”, and (**c**) “butterfly sign”.

The varying types of morphology obtained on the anterior resected surface of the femur were classified [19,20]. The vertical distances of the medial and lateral condyles between the most proximal point of the anterior bone cut on the medial and lateral condyles and the line connecting the two most distal points of the anterior bone cut on both the lateral and medial condyles were measured. The ratio of the vertical distances from the medial condyle (MD) and lateral condyle (LD) was calculated after bone resection (Figure 3). Three groups categorized based on the MD/LD ratio were as follows: group 1: <0.65 (the boot sign group), group 2: ≥0.65 and <0.8 (the grand-piano sign group), and group 3: ≥0.8 (the butterfly sign group) (Figure 4a–c).

### 2.4. Statistical Analyses

All statistical analyses were performed using SPSS for Windows (version, 19.0; SPSS, Chicago, IL, USA) and G-power (version, 3.1.5; Düsseldorf University, Germany).

For comparison of the mean values, the data were assessed for normality with the Shapiro–Wilk test. One-way ANOVA with post hoc analysis using Tukey’s honestly significant difference (HSD) test was used to compare the mean values of demographic factor, parameters (mechanical HKA, MPTA, LDFA, etc.), and morphological shape (FLAP, FMAP, etc.) among the groups and identify those factors showing statistically significant differences. The correlations between the MD/LD and variables were analyzed using the Pearson correlation coefficient to find variables correlated significant with MD/LD. Multivariate linear regression analyses with stepwise methods were used to identify variables associated with the anterior surface ratio (MD/LD ratio) and identify the group of variables that best explains the MD/LD. We accepted two-sided α-errors of 5% and β-errors of 20% to detect any significant differences. The post hoc power analysis for the primary outcome was used to determine the effect size by Cohen’s f^2^ equation using the adjusted R^2^ value in the linear regression analysis. The observed statistical power of the linear regression analysis for the MD/LD ratio was calculated to be 1.0, with an effect size of 0.3661202.

The intra- and inter-observer reliabilities of the two orthopedic surgeons were re-tested 2 weeks after the first assessment, and the average values were used. The reliability of radiological measurements was assessed by calculating the intraclass correlation coefficient (ICC).

This study was approved by our institutional review board.

## 3. Results

### 3.1. Patients’ Demographics and Parameters, Including the Morphological Shape

The overall demographics and radiological and intraoperative parameters are summarized in Table 1. The mean cTEA-PCA angle was 6.4 ± 1.8°, while the intraoperative femoral rotation angle was determined as 4.9 ± 1.2°. After femoral resection, the mean vertical distances were found to be 25.1 ± 5.8 mm (MD) and 41.6 ± 6.8 mm (LD). The overall mean MD/LD ratio was 0.61 ± 0.13, which was lower than the typical ratio of the “grand-piano sign”.

Regarding the incidence of the morphological shape, 62.4% (146/234) of patients showed incidence of the morphological shape of the “boot sign group” (MD/LD ratio = 0.65), 33.3% (78/234) showed that of the “grand-piano sign group”, and only 4.3% (10/234) exhibited the “butterfly sign group” feature.

The demographics and radiological and intraoperative parameters of the groups are summarized in Table 2. There were significant differences among the groups in FLAP, MD, LD, MD/LD ratio, and intraoperative femoral rotation angle, while the other variables showed no significant differences. In group 1, the FLAP was found to be smaller than in the other groups, while the intraoperative femoral rotation angle was found to be larger than in the other groups.

### 3.2. Factors Associated with Anterior Femoral Resectional Morphological Shape (MD/LD Ratio)

Significant correlations with the MD/LD ratio in the femoral rotation resection angle (r = −0.155, *p* = 0.018), distal femoral cutting angle (r = 0.152, *p* = 0.02), and FLAP (r = 0.176, *p* = 0.007) were observed; however, the other radiological and intraoperative parameters were not significant.

Factors associated with the MD/LD ratio obtained using multivariate linear regression analyses were FLAP, distal femoral cutting angle, and intraoperative femoral rotation angle (Table 3).

## 4. Discussion

The most important finding of this study was that the mean MD/LD ratio was 0.61 in Korean patients, and was lower than that of the typical “grand-piano sign” definition. Furthermore, the incidence of the morphological shape of the “boot sign” was found in 62.4% of cases. The MD/LD ratio was associated, especially with FLAP, indicating that the morphology of the distal femur might correlate with the MD/LD ratio.

Several authors have proposed a correlation between the shape of the anterior resection surface and femoral rotation angle in TKA [13,19,20,21]. The asymmetrical shape of the anterior resection surface of the femur, the so-called “grand-piano sign”, was considered a reference for the correct rotational angle of the femoral condylar resection when aligned parallel with TEA [13,19,20,21]. However, the shape of the anterior resection surface is known to change with the femoral rotation angle [19,20,21], distal femoral resection parameters [20,21], and even kinematic aligned TKA [20]. Cui et al. [19] have reported that the MD/LD ratio of the anterior resection surface was 0.66 and 0.69 when using the surgical TEA and 3° external rotation relative to the posterior condylar axis, respectively, by measuring the vertical distance without considering the distal femoral resection. Thus, there might be substantial differences in the vertical length and the ratio of the anterior resection surface according to the distal femoral resection, which would be more frequent in clinical practice. In a study by Ohmori et al. [21], the MD/LD ratio after distal femoral resection was 0.62 to 0.67, while the ratio increased with the increase in flexion angle of the distal femoral resection. Kim et al. [20] have reported similar results, although the MD/LD ratio in kinematically aligned TKA (range = 0.73–0.76) was found to be larger (close to the butterfly) compared with that of the mechanically aligned TKA (range = 0.57–0.63), which was lower than the definition of the “grand-piano sign”. In this study, the shape of the anterior resection surface was frequently found as a “boot sign” rather than the “grand-piano sign”. Because the parameters for the anterior resection surface were measured after all femoral bone resection were aligned mechanically, the MD/LD ratio was possibly lower than that of the “grand-piano sign” by definition, similar to previous studies [20,21]. Furthermore, the anatomical morphology of the distal femur would be different based on ethnicity or sex, which might affect the femoral rotational alignments [12,28,29,30]. Although there have been many studies on the shape of the anterior resection surface during TKA, no study has considered the distal femoral morphologies simultaneously.

Distal femoral morphology varies according to sex, ethnicity, and individual [28,29,30,31]. Previous studies have reported that the femoral posterior condyles are asymmetric in width, with the lateral side smaller than the medial [31], narrower among women than that among men [28,29,31], and longer anteroposteriorly among the Black population than that among the Asian population [30]. Furthermore, the femoral condyles in the Korean population are known to be asymmetric, as shown by larger FMAP than FLAP, which is in agreement with the Chinese population. Thus, we assumed that the shape of the anterior resection surface might be correlated with the asymmetric morphologic features of the distal femur, especially the FMAP or FLAP, and not only the femoral rotational angle. According to the results of this study, the shape of the anterior resection surface is correlated with the FLAP and femoral rotation angle, indicating that smaller FLAP is correlated with the smaller MD/LD ratio, close to the “boot sign” feature. In brief, the asymmetric distal femoral morphology, which is smaller in the lateral femoral condyle, could affect the shape of the anterior resection surface of the femur, including the femoral rotation and distal femoral cutting angles. Thus, while considering the shape of the anterior resection surface of the femur as a reference for the correct femoral rotation position, the asymmetric distal femoral morphology should be reviewed simultaneously, especially in East Asian ethnics. The intraoperative morphology of the anterior resection surface might be more frequently close to a “boot sign” owing to asymmetry. Thus, the surgeon should be careful when assessing the femoral rotation angle by the anterior resection surface, as the “boot sign” could be found frequently more than expected [20,32].

This study has several limitations. First, patients with valgus knees were excluded from this study; hence, the results might vary in patients with valgus knees. Second, the results of this study were obtained using an East Asian population; there might be differences among other ethnicities [30]. The anatomical morphology of East Asians was found to be smaller in absolute size and wider in ratio, with more asymmetric features than the Black or White population [30]; hence, there might be differences in the shape of the anterior femoral resection surface. Third, there might be differences in the ratios according to surgical techniques, such as posterior or anterior referenced TKA, and the conventional mechanical alignment or kinematic alignment during TKA [20]. Fourth, considerable individual variations based on the cartilage thickness of the femur could be observed, as well as differences owing to the use of different reference axes such as the surgical or clinical TEA and Whiteside line [24]. Finally, we did not consider the possibility that the residual cartilage of the lateral posterior femoral condyle may alter the rotational alignment. Previous studies have shown that the residual cartilage of the lateral posterior femoral condyle can change the rotation by more than 1°, although the effect on the clinical outcome of primary TKA is not significant. To overcome this limitation, we tried to remove as much of the lateral posterior femoral condyle cartilage as possible during primary TKA [15,24].

The shape of the anterior femoral resection surface was frequently found as a “boot sign” in Korean patients, which might be correlated with the asymmetric distal femoral morphology of East Asian people. Furthermore, while using the shape of the anterior resection surface of the femur as a reference for the femoral rotation angle, the distal femoral anatomical morphology, especially the FLAP and distal femoral cutting angle, should be considered simultaneously.

## 5. Conclusions

- The shape of the anterior resection surface of the femur in TKA was correlated with the femoral rotation angle, distal femoral cutting angle, and FLAP.
- Furthermore, the shape of the anterior resection surface was frequently found as a “boot sign” rather than a “grand-piano sign” in Korean ethnics owing to the asymmetric morphology of the femoral condyles.
- Differences owing to ethnicity, including distal femoral morphology, should be considered when evaluating the shape of the anterior femoral resection surface for assessment of the femoral rotation angle.

## Figures and Tables

**Table 1:** Overall patient demographics (mean ± standard deviation).

	Overall Results
Patients (number)	234
Sex (Male/Female)	44:190
Age (y)	71.9 ± 6.2
BMI	26.4 ± 4.1
Degree of osteoarthritis (Kellgren & Lawrence Score)	3.6 ± 1.2
Mechanical HKA angle	varus 9.4° ± 6.2
MPTA	84.2° ± 3.7
LDFA	82.9° ± 6.3
Coronal bowing	2.8° ± 2.6
Sagittal bowing	11.2° ± 3.4
cTEA-PCA on CT	6.4° ± 1.8
FLAP	52.9 mm ± 4.2
FMAP	53.3 mm ± 4.8
dFAP	0.3 mm ± 3.7
MD	25.1 mm ± 5.8
LD	41.6 mm ± 6.8
MD/LD ratio	0.61 ± 0.13
Distal femoral cutting angle	Valgus 5.0° ± 0.7
Intra-operative femoral rotation angle	4.9° ± 1.2

BMI: body mass index/cTEA: clinical transepicondylar axis/PCA: posterior condylar axis/HKA: Hip-Knee-Ankle axis/FLAP: femoral lateral anteroposterior length/FMAP: femoral medial anteroposterior lengths/dFAP: difference between FLAP and FMAP/FML: mediolateral femoral length/MPTA: mechanical medial proximal tibial angle/LDFA: mechanical lateral distal femoral angle.

**Table 2:** Patient demographics and parameters according to group.

	Group 1	Group 2	Group 3	*p*-Value
Patients (number)	146	78	10	-
Sex (Male/Female)	23:123	18:60	3:7	0.267
Age (y)	71.9 ± 5.9	71.9 ± 6.8	72.5 ± 7.1	0.954
BMI	26.1 ± 4.2	26.8 ± 3.9	26.5 ± 3.1	0.502
Degree of osteoarthritis (Kellgren & Lawrence Score)	3.5 ± 1.6	3.6 ± 0.6	3.6 ± 0.3	0.853
Mechanical HKA angle	9.4 ± 6.2	9.6 ± 6.4	8.3 ± 4.3	0.811
MPTA	84.3 ± 3.6	83.9 ± 3.9	85.0 ± 2.4	0.633
LDFA	83.2 ± 3.2	82.5 ± 10.0	83.7 ± 2.4	0.698
Coronal bowing	2.6 ± 2.4	3.1 ± 2.9	2.7 ± 3.0	0.469
Sagittal bowing	11.2 ± 3.2	11.4 ± 3.8	10.8 ± 3.0	0.816
cTEA-PCA on CT	6.4 ± 1.9	6.4 ± 1.7	6.1 ± 1.8	0.840
FLAP	52.4 ± 4.2	53.7 ± 4.2	54.9 ± 2.7	0.02 *
FMAP	52.7 ± 4.9	53.9 ± 4.6	55.4 ± 3.4	0.076
dFAP	0.4 ± 3.8	0.2 ± 3.7	0.5 ± 2.9	0.933
MD	22.6 ± 4.9	28.7 ± 4.4	32.5 ± 7.1	<0.001 *
LD	42.8 ± 6.7	39.8 ± 6.2	37.4 ± 9.8	<0.001 *
MD/LD ratio	0.53 ± 0.07	0.72 ± 0.04	0.89 ± 0.11	<0.001 *
Distal femoral cutting angle	4.9 ± 0.7	5.1 ± 0.6	5.1 ± 0.3	0.112
Intra-operative femoral rotation angle	5.0 ± 1.2	4.6 ± 1.1	4.7 ± 1.2	0.039 *

The ICC values for radiographic measurements ranged from 0.75 to 0.93, indicating good-to-excellent agreement. * The difference between the groups was significant (*p* < 0.05).

**Table 3:** Association of factors with MD/LD ratio in multivariate linear regression analysis.

Factor	Mean		
	β ± SE	*p*-Value	Adjusted R^2^
Intercept	0.304		
FLAP	0.005 ± 0.002	0.016	0.268
Distal femoral cutting angle	0.025 ± 0.012	0.044	
Intra-operative femoral rotation angle	−0.105 ± 0.08	0.037	

## Data Availability

No new data were created or analyzed in this study. Data sharing is not applicable to this article.

## References

[1] Kim K., Kim J., Lee D., Lim S., Eom J. (2019). The Accuracy of Alignment Determined by Patient-Specific Instrumentation System in Total Knee Arthroplasty. Knee Surg. Relat. Res..

[2] Ko D.O., Lee S., Kim J.H., Hwang I.C., Jang S.J., Jung J. (2021). The Influence of Femoral Internal Rotation on Patellar Tracking in Total Knee Arthroplasty using Gap Technique. Clin. Orthop. Surg..

[3] Choi Y.J., Seo D.K., Lee K.W., Ra H.J., Kang H.W., Kim J.K. (2020). Results of total knee arthroplasty for painless, stiff knees. Knee Surg. Relat. Res..

[4] Goto K., Katsuragawa Y., Miyamoto Y. (2020). Outcomes and component-positioning in total knee arthroplasty may be comparable between supervised trained surgeons and their supervisor. Knee Surg. Relat. Res..

[5] Song S.J., Kim K.I., Suh D.U., Park C.H. (2021). Comparison of Patellofemoral-Specific Clinical and Radiographic Results after Total Knee Arthroplasty Using a Patellofemoral Design-Modified Prosthesis and Its Predecessor. Clin. Orthop. Surg..

[6] Armstrong A.D., Brien H.J., Dunning C.E., King G.J., Johnson J.A., Chess D.G. (2003). Patellar position after total knee arthroplasty: Influence of femoral component malposition. J. Arthroplast..

[7] Choi Y., Koo J., Moon S.W., Yang Y., Son J. (2020). Long-term Follow-up of Patellar Nonresurfacing in Total Knee Arthroplasty. Clin. Orthop. Surg..

[8] Abdelnasser M.K., Elsherif M.E., Bakr H., Mahran M., Othman M.H.M., Khalifa Y. (2019). All types of component malrotation affect the early patient-reported outcome measures after total knee arthroplasty. Knee Surg. Relat. Res..

[9] Chon J., Jeon T., Yoon J., Jung D., An C.H. (2019). Influence of Patellar Tilt Angle in Merchant View on Postoperative Range of Motion in Posterior Cruciate Ligament-Substituting Fixed-Bearing Total Knee Arthroplasty. Clin. Orthop. Surg..

[10] Siston R.A., Patel J.J., Goodman S.B., Delp S.L., Giori N.J. (2005). The variability of femoral rotational alignment in total knee arthroplasty. J. Bone Joint Surg. Am..

[11] Satit T., Pinyong U., Chaipipathn S., Natthapong H., Revit T. (2021). Imageless robotic-assisted total knee arthroplasty accurately restores the radiological alignment with a short learning curve: A randomized controlled trial. Int. Orthop..

[12] Victor J. (2009). Rotational alignment of the distal femur: A literature review. Orthop. Traumatol. Surg. Res..

[13] Skowronek P., Arnold M., Starke C., Bartyzel A., Moser L.B., Hirschmann M.T. (2021). Intra- and postoperative assessment of femoral component rotation in total knee arthroplasty: An EKA knee expert group clinical review. Knee Surg. Sports Traumatol. Arthrosc..

[14] Asada S., Akagi M., Matsushita T., Hashimoto K., Mori S., Hamanishi C. (2012). Effects of cartilage remnants of the posterior femoral condyles on femoral component rotation in varus knee osteoarthritis. Knee.

[15] Tashiro Y., Uemura M., Matsuda S., Okazaki K., Kawahara S., Hashizume M., Iwamoto Y. (2012). Articular cartilage of the posterior condyle can affect rotational alignment in total knee arthroplasty. Knee Surg. Sports Traumatol. Arthrosc..

[16] Matziolis D., Meiser M., Sieber N., Teichgraber U., Matziolis G. (2017). Posterior Cortical Axis: A New Landmark to Control Femoral Component Rotation in Total Knee Arthroplasty. Orthopedics.

[17] Goki K., Shigeki I., Koki Y., Satoru S., Hiroyuki I., Masakazu I., Yu M., Nobuo A. (2021). Accuracy of total knee arthroplasty using the modified gap technique based on the bone gap: An evaluation of the bone gap with a distal femoral trial component. J. Arthroplast..

[18] Silva A., Pinto E., Sampaio R. (2016). Rotational alignment in patient-specific instrumentation in TKA: MRI or CT?. Knee Surg. Sports Traumatol. Arthrosc..

[19] Cui W.Q., Won Y.Y., Baek M.H., Kim K.K., Cho J.H. (2006). Variations of the ‘grand-piano sign’ during total knee replacement. A computer-simulation study. J. Bone Joint Surg. Br..

[20] Kim J.T., Han J., Shen Q.H., Moon S.W., Won Y.Y. (2018). Morphological Patterns of Anterior Femoral Condylar Resection in Kinematically and Mechanically Aligned Total Knee Arthroplasty. J. Arthroplast..

[21] Ohmori T., Kabata T., Kajino Y., Taga T., Inoue D., Yamamoto T., Takagi T., Yoshitani J., Ueno T., Tsuchiya H. (2018). Usefulness of the “grand-piano sign” for determining femoral rotational alignment in total knee arthroplasty. Knee.

[22] Moser L.B., Hess S., Bargemon J.B., Faizan A., LiArno S., Amsler F., Hirschmann M.T., Ollivier M. (2022). Ethnical Differences in Knee Phenotypes Indicate the Need for a More Individualized Approach in Knee Arthroplasty: A Comparison of 80 Asian Knees with 308 Caucasian Knees. J. Pers. Med..

[23] Song K., Ran T., Ma T., Qin Y., Zhang B., Wang M. (2023). A Morphometric Study of the Distal Femoral Resected Surface In Osteoarthritis Knees of the Patients in Southwest China and a Comparison with Femoral Components in Six Total Knee Arthroplasty Systems. Orthop. Surg..

[24] Kim S.H., Park Y.B., Ham D.W., Lee J.S., Song M.K., Lee H.J. (2017). No influence of femoral component rotation by the lateral femoral posterior condylar cartilage remnant technique on clinical outcomes in navigation-assisted TKA. Knee Surg. Sports Traumatol. Arthrosc..

[25] Ko J.H., Han C.D., Shin K.H., Nguku L., Yang I.H., Lee W.S., Kim K.L., Park K.K. (2016). Femur bowing could be a risk factor for implant flexion in conventional total knee arthroplasty and notching in navigated total knee arthroplasty. Knee Surg. Sports Traumatol. Arthrosc..

[26] Akamatsu Y., Kobayashi H., Kusayama Y., Kumagai K., Saito T. (2016). Femoral shaft bowing in the coronal and sagittal planes on reconstructed computed tomography in women with medial compartment knee osteoarthritis: A comparison with radiograph and its predictive factors. Arch. Orthop. Trauma. Surg..

[27] Kim S.H., Lee H.J., Jung H.J., Lee J.S., Kim K.S. (2013). Less femoral lift-off and better femoral alignment in TKA using computer-assisted surgery. Knee Surg. Sports Traumatol. Arthrosc..

[28] Bellemans J., Carpentier K., Vandenneucker H., Vanlauwe J., Victor J. (2010). The John Insall Award: Both morphotype and gender influence the shape of the knee in patients undergoing TKA. Clin. Orthop. Relat. Res..

[29] Kim J.B., Lyu S.J., Kang H.W. (2016). Are Western Knee Designs Dimensionally Correct for Korean Women? A Morphometric Study of Resected Femoral Surfaces during Primary Total Knee Arthroplasty. Clin. Orthop. Surg..

[30] Kim T.K., Phillips M., Bhandari M., Watson J., Malhotra R. (2017). What Differences in Morphologic Features of the Knee Exist Among Patients of Various Races? A Systematic Review. Clin. Orthop. Relat. Res..

[31] Brzobohatá H., Krajíček V., Horák Z., Velemínská J. (2016). Sexual Dimorphism of the Human Tibia through Time: Insights into Shape Variation Using a Surface-Based Approach. PLoS ONE.

[32] Kokubu Y., Kawahara S., Hamai S., Akasaki Y., Tsushima H., Miyachika S., Nakashima Y. (2023). “Grand-piano sign” as a femoral rotational indicator in both varus and valgus knees: A simulation study of anterior resection surface in total knee arthroplasty. Knee Surg. Sports Traumatol. Arthrosc..

